# Multiple Sclerosis Autoantigen Myelin Basic Protein Escapes Control by Ubiquitination during Proteasomal Degradation*

**DOI:** 10.1074/jbc.M113.544247

**Published:** 2014-04-16

**Authors:** Alexey Belogurov, Anna Kudriaeva, Ekaterina Kuzina, Ivan Smirnov, Tatyana Bobik, Natalia Ponomarenko, Yelena Kravtsova-Ivantsiv, Aaron Ciechanover, Alexander Gabibov

**Affiliations:** From the ‡ Shemyakin-Ovchinnikov Institute of Bioorganic Chemistry, Russian Academy of Sciences, 117871 Moscow V-437, Russia,; the § Institute of Gene Biology, Russian Academy of Sciences, 117334 Moscow, Russia,; the ¶ Department of Chemistry, Lomonosov Moscow State University, Moscow 119991, Russia, and; the ‖ Cancer and Vascular Biology Center, The Rappaport Faculty of Medicine and Research Institute, Technion-Israel Institute of Technology, Haifa 31096, Israel

**Keywords:** Antigen Processing, Autoimmune Disease, Multiple Sclerosis, Myelin, Oligodendrocyte, Proteasome, Ubiquitin

## Abstract

The vast majority of cellular proteins are degraded by the 26S proteasome after their ubiquitination. Here, we report that the major component of the myelin multilayered membrane sheath, myelin basic protein (MBP), is hydrolyzed by the 26S proteasome in a ubiquitin-independent manner both *in vitro* and in mammalian cells. As a proteasomal substrate, MBP reveals a distinct and physiologically relevant concentration range for ubiquitin-independent proteolysis. Enzymatic deimination prevents hydrolysis of MBP by the proteasome, suggesting that an abnormally basic charge contributes to its susceptibility toward proteasome-mediated degradation. To our knowledge, our data reveal the first case of a pathophysiologically important autoantigen as a ubiquitin-independent substrate of the 26S proteasome.

## Teaser

**Background:** Most proteins must be ubiquitinated prior to proteasomal degradation.

**Results:** Myelin basic protein (MBP) is hydrolyzed by the 26S proteasome without ubiquitination *in vitro* and in mammalian cells.

**Conclusion:** Proteasome-mediated hydrolysis of the multiple sclerosis autoantigen MBP is uncontrolled by the ubiquitination system.

**Significance:** Results reveal the first example of an autoantigen degraded by the proteasome without ubiquitin.

## Introduction

Myelin basic protein (MBP)^3^ is one of the key structural elements of the myelin sheath, which covers axons to significantly enhance rapid conduction of nerve impulses. Interestingly, MBP, in addition to the myelin oligodendrocyte glycoprotein, is also one of the major autoantigens identified in multiple sclerosis (MS). MBP and its peptides have been extensively studied for decades as important components of the autoimmune response and encephalitogenic agents (1, 2), potential MS drugs (3, 4), and substrates for degradation by enzymes and abzymes (5, 6). Therefore, it is obvious that the metabolism of MBP is important for myelin biogenesis in both health and disease.

The ubiquitin proteasome system is implicated in a number of neurodegenerative diseases, including Parkinson, Alzheimer, Huntington, prion diseases, and amyotrophic lateral sclerosis, as reviewed by Ciechanover and Brundin (7). MS, similar to its animal model-experimental autoimmune encephalomyelitis (8), is a severe disorder of the central nervous system (CNS), accompanied by dramatic failures in numerous metabolic and regulating cellular pathways, which are largely associated with the immune system (9). Recent findings indicate that the proteasome, in its constitutive and immuno states, may play an important role in MS (10). Previously, Goverman and colleagues (11) showed that myelin-specific CD8^+^ cytotoxic lymphocytes induce severe CNS autoimmunity in mice. The 26S proteasome is the major supplier of MHC class I-associated peptides, which are responsible for the recognition of the cell by CD8^+^ cytotoxic lymphocytes (12). Recent studies indicate that the peptides generated by the proteasome may also be exposed on MHC class II molecules (13). Therefore, this processive molecular machine has to be strictly controlled in the cell to ensure that the peptides exposed on the cell surface do not cause an autoimmune reaction but instead mark infected or transformed cells. The ubiquitination system has evolved to orchestrate the life cycle of a large part of the cell proteome because an absolute majority of cellular proteins are recognized by the 26S proteasome only after ubiquitination. To date, there are a number of cases that describe proteasome-mediated proteolysis without ubiquitin (14). Previous studies have reported that ornithine decarboxylase (ODC) (15), p21 (16), and FAT10 (17) are hydrolyzed by the 26S proteasome through a ubiquitin-independent mechanism. The recently reported ubiquitin-independent degradation of acetylated core histones is proposed to be mediated by bromodomain-like regions of the proteasome activator PA200 (18). The majority of other studies provide evidence that the 20S proteasome can degrade proteins in a ubiquitin-independent manner (reviewed in Ref. 19). However, the role that the 20S proteasome has in cellular proteolysis, if any, is controversial. In the present study, we addressed whether MBP is a substrate for the proteasome-ubiquitin machinery and, if so, how the ubiquitination system controls its degradation. Our data suggest that the 26S-mediated degradation of intracellular MBP is ubiquitin-independent.

## EXPERIMENTAL PROCEDURES

### 

#### 

##### Plasmid Construction

Human MBP cDNA was assembled using direct hybridization of overlapping oligonucleotides, which covered the whole targeted sequence, and subsequent amplification by polymerase chain reaction. Further MBP cDNAs were amplified using appropriate primers with flanking NcoI/XhoI and KpnI/XhoI sites, and the PCR products were subcloned into modified pET22N-FLAG (prokaryotic expression and *in vitro* translation) and pBudCE4.1/EF-FLAG (20) (mammalian expression) plasmids, respectively. The cDNAs coding for HA-Ub, myc-Ub, myc-UbK0, and HA-c-Myc were kindly provided by Dr. Kazuhiro Iwai and further subcloned into pCAGGS for expression in mammalian cells. The cDNAs coding for human p105, which were used for *in vitro* translation (in pT7b105) and transient transfections in HEK293 cells (pFLAG-CMV2), were described previously (21). The cDNAs coding for ODC-FLAG and antizyme-FLAG were kindly provided by Dr. Chaim Kahana. The pmaxGFP vector (Lonza) was used for mammalian GFP expression.

##### Ethics Statement

BALB/c mice were developed in significant pathogen-free conditions in the Pushchino branch of the Shemyakin and Ovchinnikov Institute of Bioorganic Chemistry, Russian Academy of Sciences, in accordance with the regulations of the Department of Health and Human Services, National Institutes of Health Animal Welfare Insurance.

##### Cultured Cells and Transfection Procedures

HEK293 or HeLa cells were grown at 37 °C and 5% CO_2_ in DMEM supplemented with 10% fetal calf serum and antibiotics (penicillin-streptomycin). Mature murine ODC cultures were prepared from brains of 3-day-old SJL mouse pups as described in Ref. 22. The cDNA and siRNA transfections were accomplished using Lipofectamine LTX with Plus reagent and the Lipofectamine siRNA Max kit, respectively (Invitrogen). Alternatively, the cDNAs/siRNAs were transfected using the Nucleofector device (Lonza) with the “transfection efficiency” program. All ofthe procedures were performed according to the manufacturer's instructions.

##### Cycloheximide Chase Experiments

To study the proteasomal degradation of MBP, ODC, and c-Myc proteins in HEK293 cells, cycloheximide (100 μg/ml) was added to transfected cells for the indicated times, and the cells were lysed using radioimmune precipitation assay buffer (150 mm NaCl, 0.5% sodium deoxycholate, 50 mm Tris-HCl, pH 8, 0.1% SDS, 1% Nonidet P-40, and protease inhibitor mixture). Protein lysates prepared from an equal number of cells were resolved via SDS-PAGE and blotted onto nitrocellulose membranes. MBP and ODC were visualized using an anti-FLAG antibody, and c-Myc was visualized using an anti-HA antibody. Actin was detected using a specific antibody.

##### Stability of MBP, ODC, and c-Myc in Cells in the Presence of K0/WT Ubiquitin Species

HEK293 cells that were 70% confluent in a six-well plate were transiently transfected with 3 μg of cDNA coding for WT or K0 Myc-tagged ubiquitins. After 24 h, the cells were transiently transfected with 1 μg of cDNA coding for MBP-FLAG, ODC-FLAG, or HA-c-Myc. After an additional 24 h, the cells were subjected to cycloheximide treatment.

##### In Vivo Ubiquitination

HEK293 cells were transiently transfected as described with the cDNA coding for MBP-FLAG, FLAG-p105, and Ubc6 w/o FLAG epitope and the cDNAs coding for WT HA-tagged ubiquitin. After 24 h, the proteasome inhibitor MG132 (20 μm) was added for 2.5 h, and the cells were lysed with radioimmune precipitation assay buffer supplemented with freshly dissolved iodoacetamide and *N*-ethylmaleimide (5 mm each) to inhibit the deubiquitinating enzymes. The proteins were immunoprecipitated with immobilized anti-FLAG antibody and subjected to Western blotting analysis. The proteins were visualized using anti-FLAG and anti-HA antibodies.

##### siRNA Gene Silencing

HEK293 cells that were 60% confluent in a six-well plate were transfected with 100 pmol of either targeting or non-targeting siRNAs (Dharmacon, Thermo Scientific). After 24 h, the cells were transfected with 1 μg of cDNAs coding for tested proteins. After an additional 24 h, the cells were subjected to cycloheximide treatment.

##### Purification of the Proteasome from Mouse Brains

Briefly, a BALB/c brain was homogenized using a dounce homogenizer into three parts w/w lysis buffer containing 30 mm Tris-HCl (pH 7.5), 2 mm ATP, 1 mm EDTA, 5 mm MgCl_2_, 1 mm DTT, 10% glycerol, 100 mm NaCl, and a protease inhibitor mixture. The prepared brain homogenate was subjected to three repeated freeze-thaw cycles, and further cell debris was removed by two consecutive centrifugations at 4 °C (1,500 × *g* for 20 min and 13,000 × *g* for 30 min). The supernatant (0.8 ml) was overlaid on top of a 24-ml 10–55% glycerol gradient in 25 mm Tris-HCl (pH 7.5), 1 mm DTT, and 4 mm ATP and centrifuged at 125,000 × *g*, 4 °C for 16 h. The fractions (1 ml each) were collected, and the proteasome activity was quantified using Suc-LLVY-MCA as a substrate. To distinguish between the activity related to the 20S proteasome and that related to the 26S proteasome, the assay was performed with or without 0.02% SDS. The buffer used to measure the activity of the proteasomes contained 20 mm Tris, pH 7.5, 1 mm ATP, 1 mm DTT, and 5 mm MgCl_2_. The fractions containing the 26S proteasome were subjected to ion-exchange chromatography on a MonoQ column using an NaCl gradient (100–500 mm in 15 column volumes) in buffer containing 20 mm Tris (pH 7.5), 1 mm ATP, 1 mm DTT, and 5 mm MgCl_2_. The fractions containing the 26S proteasome were dialyzed into storage buffer (25 mm Tris-HCl (pH 7.5), 1 mm DTT, 1 mm ATP, 5 mm MgCl_2_, and 10% glycerol). For long term storage, up to 40% glycerol was added to the proteasome, and the further purified proteasome was stored at −20 °C for two months.

##### In Vitro Translation

The rhMBP and p105 proteins were translated *in vitro* in the presence of l-[^35^S]methionine using the TnT T7 quick reticulocyte lysate-based coupled transcription-translation kit (Promega) according to the manufacturer's instructions.

##### Recombinant Protein Expression and Purification

The recombinant human MBP was expressed in *Escherichia coli* BL21(DE3) cells. The transformed cells were grown to a density *A*_600_ of 0.5–0.6 at 37 °C. Isopropyl β-d-1-thiogalactopyranoside was added to the cells to obtain a final concentration of 1 mm. After 30 min, rifampicin (200 μg/ml) was added, and the cells were further incubated for 1 h at 42 °C. The primary isolation of MBP was accomplished by metal-chelate chromatography using a nickel-nitrilotriacetic acid column under denaturing conditions (6 m guanidine chloride). Next, MBP was extracted from the eluates by chloroform-methanol (1:3), precipitated by saturated ammonium sulfate, and reprecipitated by cold acetone (9 v/v) in the presence of 1 mm HCl. The final purification was performed using reversed-phase HPLC on a C4 column.

##### Preparation and Fractionation of Crude Reticulocyte Lysate

Reticulocytes were induced in rabbits, and the lysates were prepared and fractionated over DEAE cellulose onto fractions I and II, as described previously (23).

##### In Vitro MBP Hydrolysis and Conjugation

The hydrolysis or conjugation of proteins was performed in a volume of 12.5 μl. The reaction mixtures contained 20 mm Tris (pH 7.5), 1 mm DTT, 5 mm MgCl_2_, and the following components where indicated: fraction II (60 μg), HeLa, or BALB/c extract (60 μg), ubiquitin species (5.0 μg), E1 (0.25 μg), bacterially expressed His_6_-tagged UbcH5c (0.75 μg), ATPγS (2.0 mm), ATP (1.0 mm), and ATP-regenerating system (phosphocreatine (10 mm) and creatine phosphokinase (0.5 μg)), UbAl (0.5 μg), MG132 (200 μm), PS-341 (1.0 μm), and purified 26S proteasomes (0.25 μg). The mixtures were incubated for the indicated time at 37 °C, and the reaction was terminated by the addition of sample buffer. The proteins were resolved by SDS-PAGE and visualized via different methodologies depending on the initial substrate concentration: ^35^S-labeled proteins (10,000 cpm), PhosphorImager (Fuji), 0.1 μg of protein Western blotting, and 1.5 μg of protein Coomassie staining. If required, ATP was depleted from the proteasome samples by incubation with 50 units/ml hexokinase in buffer containing 20 mm glucose and 5 mm MgCl_2_ for 30 min at 37 °C.

##### Chemical Acetylation and Enzymatic Deamination

Myelin basic protein was acetylated and deiminated as described by Monferran *et al.* (24) and by Pritzker *et al.* (25), respectively.

##### Native and Basic Native PAGE

Native and basic native PAGE were performed according to the protocols described by Elsasser *et al.* (26) and Reisfeld *et al.* (27), respectively.

## RESULTS

### 

#### 

##### Proteasomal Degradation of MBP in Mammalian Cells Does Not Require Ubiquitination

**FIGURE 1.**
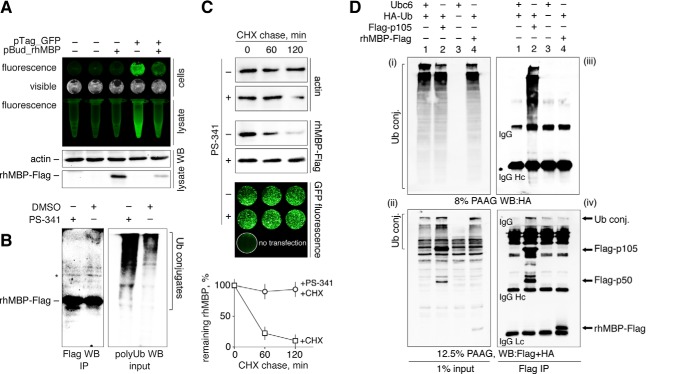
**Myelin basic protein is degraded by proteasomes in mammalian cells but shows no detectable *in vivo* ubiquitination.** *A*, HEK293 cells were transfected with cDNAs coding for rhMBP and GFP. After 24 h, cells were checked for GFP fluorescence, lysed, and subjected to Western blot analysis (*WB*). *B*, HEK293 cells were transfected with cDNA coding for rhMBP-FLAG. After 24 h, cells were treated with PS-341 or dimethyl sulfoxide for 3 h, lysed, and subjected to FLAG immunoprecipitation followed by Western blot analysis. The eluates were stained for the FLAG epitope (IP, *left*), and the input was stained with anti-polyUb antibodies (*right panel*) to verify the accumulation of polyubiquitinated conjugates (*conj.*). An artificial band existing in all MBP samples is marked by an *asterisk. C*, HEK293 cells were transfected with cDNAs coding for recombinant human MBP and GFP. After 24 h, the cells were subjected to cycloheximide chase in the presence or absence of the proteasome inhibitor PS-341. The percentage of protein remaining was calculated as the ratio of protein at the time points indicated relative to the initial protein. The data are represented as the mean ± S.E. from three separate experiments. *D*, HEK293 cells were co-transfected with cDNAs coding for control Ubc6 without FLAG epitope and HA-Ub (1), p105-FLAG and HA-Ub (2), Ubc6 alone (3), or MBP-FLAG and HA-Ub (4). After 24 h, the cells were treated with MG132 for 2.5 h, lysed, and subjected to FLAG immunoprecipitation followed by Western blotting analysis. One percent of the total cell lysates (*i–ii*) and immunoprecipitates (*iii–iv*) were stained for HA tag (*i–iii*) and for HA and FLAG tag simultaneously (*ii–iv*). *IP*, immunoprecipitation; *CHX*, cycloheximide; *PAAG*, polyacrylamide gel; *DMSO*, dimethyl sulfoxide.

The intracellular degradation of MBP was tested in HEK293 cells transfected with cDNAs coding for the rhMBP and GFP proteins (Fig. 1*A*). We immunoprecipitated MBP from transfected HEK293 cells either treated or not treated with the proteasome inhibitor bortezomib (PS-341) (Fig. 1*B*). Western blotting analysis showed that MBP was not ubiquitinated, despite the accumulation of other polyubiquitinated proteins in the cells treated with the proteasome inhibitor. Next, the transfected cells were treated with cycloheximide in the presence or absence of PS-341 and further subjected to Western blotting analysis (Fig. 1*C*). The amount of rhMBP in total cell lysates rapidly decreased following the addition of cycloheximide, which is in contrast to the behavior of the cells subjected to cycloheximide and PS-341. This finding suggests that rhMBP is degraded by the proteasome *in vivo*. The amount of GFP protein, which is not a substrate for the proteasome (28), did not significantly change in cells subjected to cycloheximide with or without PS-341 treatment.

If a protein is to be degraded through proteasome-mediated hydrolysis, it normally undergoes modification by ubiquitin (Ub) prior to its recognition by the 26S proteasome (14). To determine whether MBP is ubiquitinated *in vivo*, rhMBP-FLAG was immunoprecipitated from cells that were co-transfected with cDNAs coding for rhMBP and HA-tagged ubiquitin (Fig. 1*D*). HA-Ub is incorporated into the newly formed polyubiquitin chains and can then be detected through Western blotting techniques. The FLAG-tagged NF-κB precursor protein p105, which undergoes ubiquitin conjugation (21), was used as a positive control. After treatment with the proteasome inhibitor MG132, we failed to detect any polyubiquitin conjugates in the precipitates containing MBP, in contrast to the positive results obtained with the control p105 protein. Taken together, these data suggest that, in mammalian cells, MBP is subjected to proteasomal degradation, most likely in the absence of polyubiquitination.

##### Proteasomal Degradation of MBP in Mammalian Cells Is Not Affected by UbK0 Co-transfection and E1 Silencing

**FIGURE 2.**
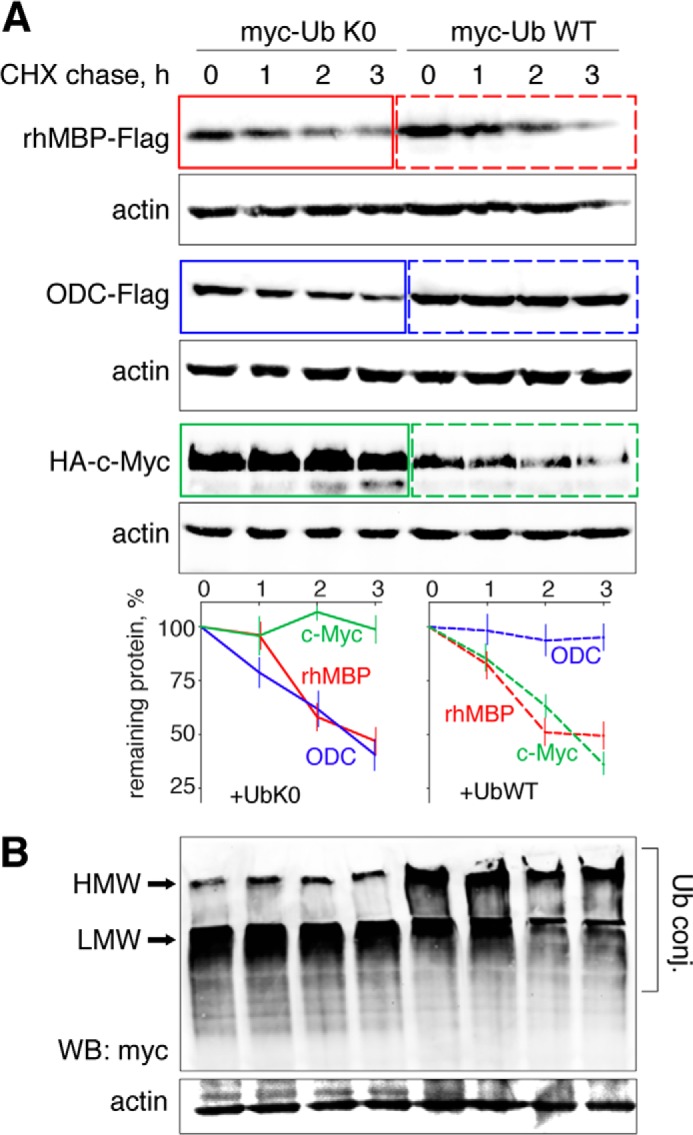
**Proteasome-mediated degradation of MBP in mammalian cells is not affected by UbK0 co-transfection.** *A*, HEK293 cells were transfected with cDNAs coding for MBP, ODC, and c-Myc, along with cDNAs coding for the respective ubiquitin species. After 24 h, the cells were subjected to cycloheximide (*CHX*) chase followed by Western blotting (*WB*) analysis. The percentage of protein remaining was calculated as the ratio of protein at the time points indicated relative to the initial protein. The data are represented as the mean ± S.E. from three separate experiments. *B*, the Ub K0/WT-transfected samples were additionally stained for Myc tag to visualize the accumulation of low molecular weight (*LMW*) and high molecular weight (*HMW*) polyubiquitin conjugates, which are marked by *arrows. Ub conj.*, ubiquitin conjugates.

**FIGURE 3.**
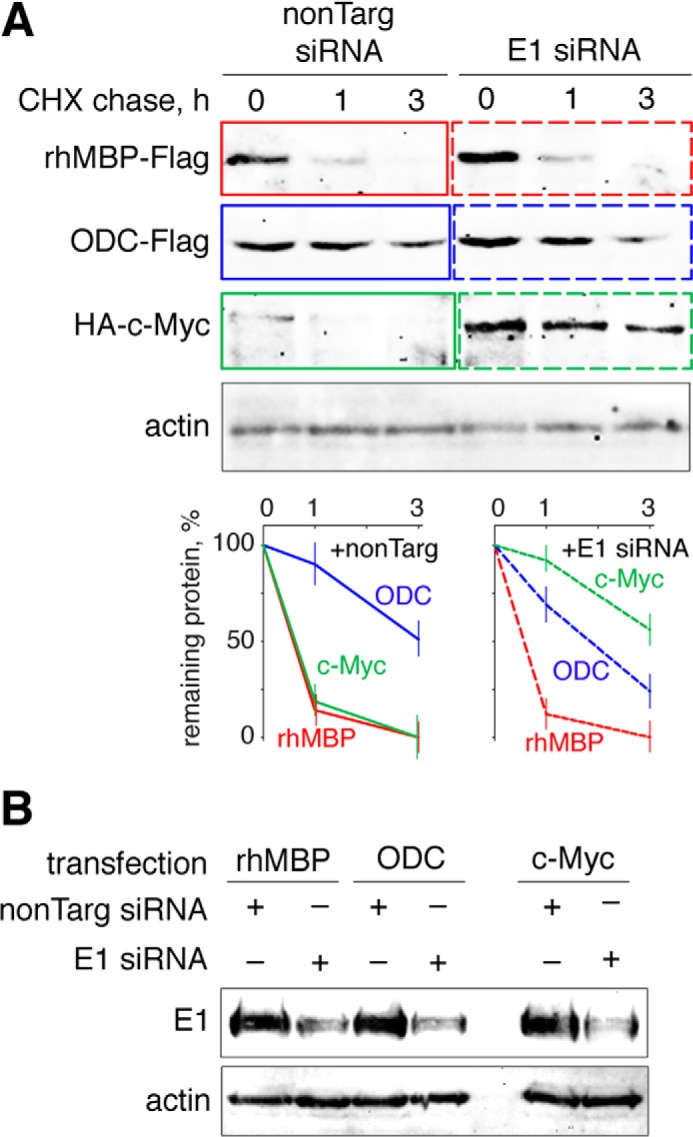
**Proteasome-mediated degradation of MBP in mammalian cells is not affected by E1 silencing.** *A*, HEK293 cells were transfected with cDNAs coding for MBP, ODC, and c-Myc, in addition to either non-targeting or anti-E1 siRNA. After 24 h, the cells were subjected to cycloheximide (*CHX*) chase followed by Western blotting analysis. The percentage of protein remaining was calculated as the ratio of protein at the time points indicated relative to the initial protein. The data are represented as the mean ± S.E. from three separate experiments. *B*, the knockdown of E1 in treated cells compared with control cells was confirmed by staining for the E1 protein. *nonTarg*, non-targeting.

**FIGURE 4.**
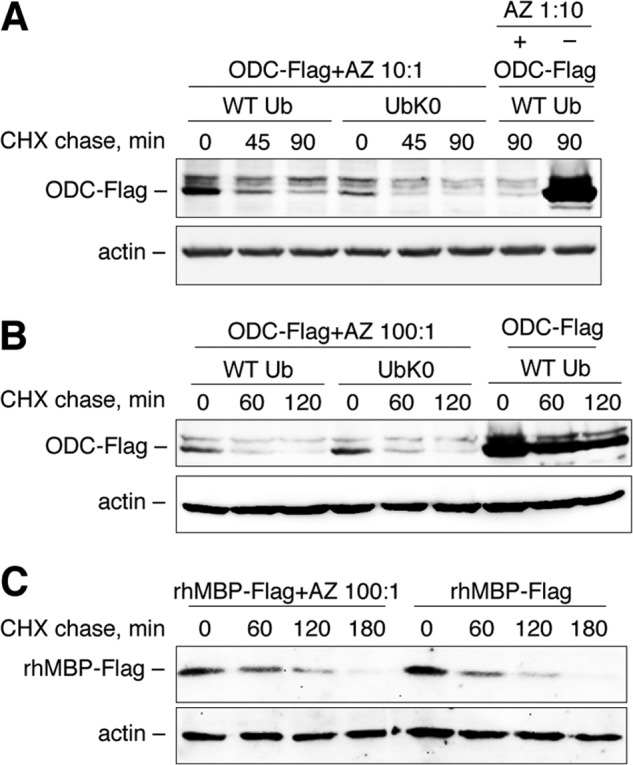
**Proteasome-mediated degradation of MBP in mammalian cells is not affected by antizyme.** HEK293 cells were transfected with cDNAs coding for ODC or rhMBP, in addition to cDNAs coding for the respective ubiquitin species, as indicated. cDNAs coding for antizyme (*AZ*) were co-transfected with ODC at different antizyme:protein ratios: 1:10 (*A*) and 1:100 (*B* and *C*). After 24 h, the cells were treated by cycloheximide (*CHX*), as indicated and subjected to Western blotting analysis.

To test whether polyubiquitination is required for MBP hydrolysis in mammalian cells, we performed a set of experiments involving Myc-tagged ubiquitin K0 (UbK0) and siRNA targeting of E1. UbK0 is a non-polymerizable variant of wild-type ubiquitin that lacks lysines and prevents the growth of polyubiquitin chains (21). E1 is known as a ubiquitin-activating enzyme and is the primary protein in the hierarchy of the ubiquitin machinery (14). Therefore, HEK293 cells were transfected with cDNAs coding for either wild-type or K0 ubiquitins (Fig. 2). Alternatively, cells were transfected with non-targeting or E1-targeting siRNA (Fig. 3). Twenty-four hours later, the cells were transfected with cDNAs coding for rhMBP, c-Myc, or ODC proteins and further subjected to a cycloheximide chase. We chose c-Myc and ODC as distinct functional controls because the proteasomal degradation of c-Myc is strictly dependent on ubiquitination (29), whereas ODC is hydrolyzed by the proteasome in the presence of the antizyme protein and without ubiquitin (15). As anticipated, co-transfection of UbK0 or E1 siRNA, compared with UbWT and non-targeting siRNA, respectively, inhibited proteasomal hydrolysis and triggered the accumulation of c-Myc protein. In contrast, the degradation of ODC was independent of the ubiquitin machinery. Moreover, compared with c-Myc protein, ODC accumulated to a lesser degree and degraded significantly faster in cells transfected with UbK0 in the absence of exogenous antizyme, most likely due to the lack of competitive polyubiquitinated substrates. Antizyme strongly accelerated ODC hydrolysis, whereas it had no detectable influence on MBP degradation (Fig. 4). Cells co-transfected with cDNAs coding for the antizyme, together with ODC (antizyme:ODC cDNA ratio 1:100), significantly decreased the amount of ODC from the start of the experiment, without any addition of cycloheximide and regardless of the absence or presence of UbK0. In line with our previous observations, MBP was degraded by proteasomes under all conditions, regardless of the disarray of the ubiquitination system.

**FIGURE 5.**
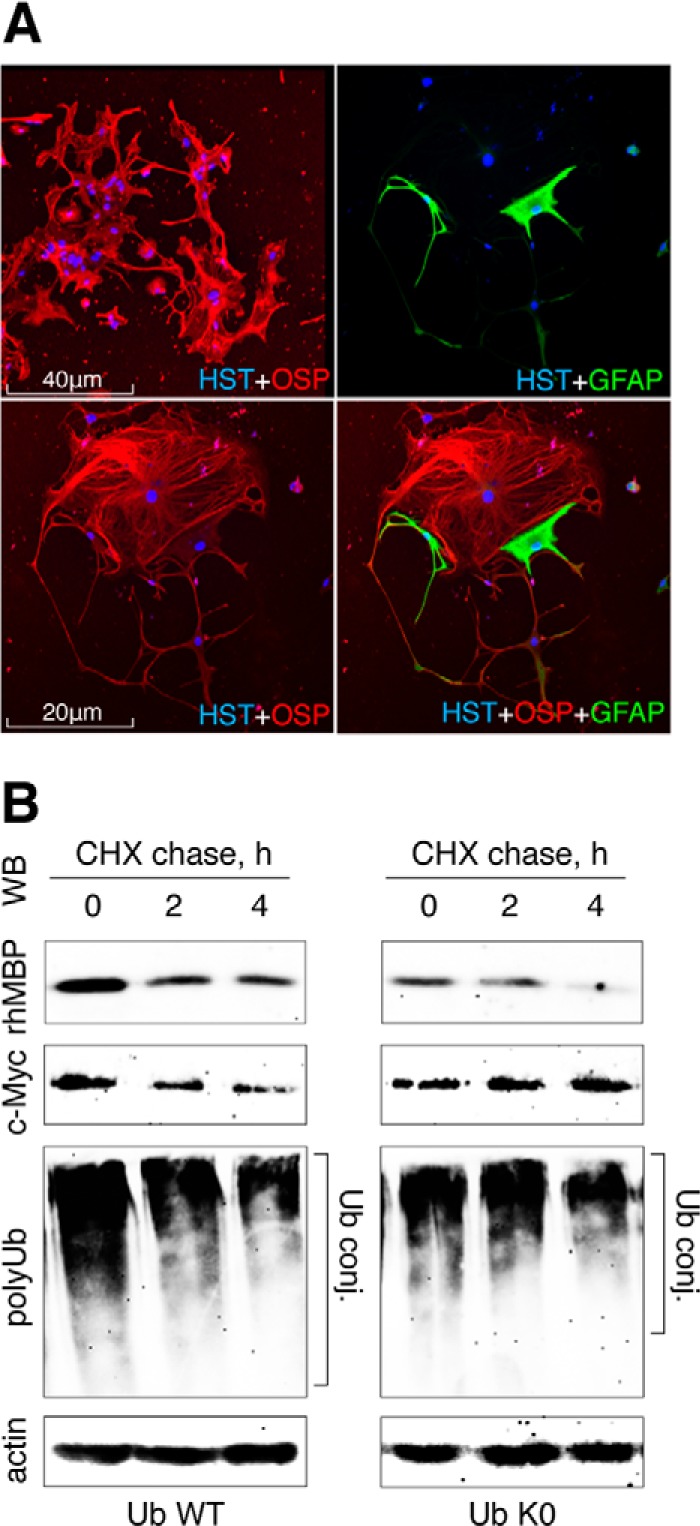
**Proteasome-mediated degradation of MBP in mature oligodendrocytes is not affected by UbK0 co-transfection.** *A*, panels represent double immunostaining of mature oligodendrocytes for oligodendrocyte-specific protein (*OSP*) and glial fibrillary acidic protein (*GFAP*), as indicated. *HST*, Hoechst. *B*, oligodendrocytes were transfected with cDNAs coding for rhMBP-FLAG or c-Myc-9HA, along with cDNAs coding for the respective ubiquitin species. After 36 h, the cells were subjected to cycloheximide (*CHX*) chase followed by Western blotting (*WB*) analysis. The data shown are representative of three separate experiments. *conj.*, conjugates.

To investigate proteasomal degradation of MBP in cells professionally maintained with MBP, we used mature murine oligodendrocytes. Cultured cells predominantly expressed oligodendrocyte-specific protein, whereas staining for glial fibrillary acidic protein, a marker of actrocytes, was observed in a significantly lesser extent (Fig. 5*A*). Oligodendrocytes were transiently co-transfected with cDNA coding for rhMBP or c-Myc, together with cDNA coding for UbWT and UbK0. Similar to experiments involving HEK293 cells, rhMBP was degraded by proteasomes at the same rate or an even faster rate in the presence of non-polymerizable UbK0 in comparison with wild type Ub (Fig. 5*B*). In contrast, degradation of c-Myc and polyUb conjugates was inhibited in the presence of UbK0. Taken together, our data revealed that MBP, similar to ODC, can be a proteasome substrate in mammalian cells without prior ubiquitination.

##### Proteasomal Degradation of MBP at Micromolar Concentrations Escapes Control by Ubiquitination

**FIGURE 6.**
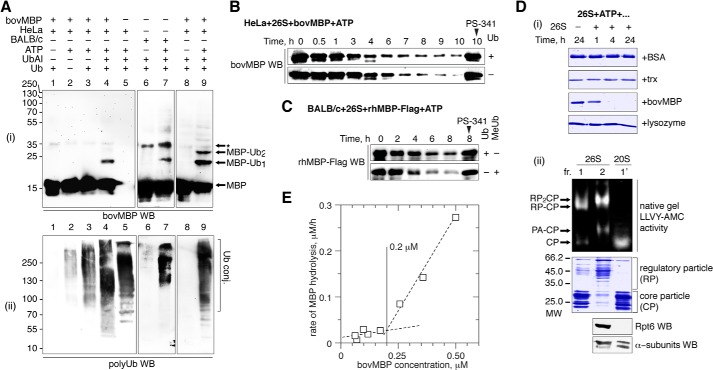
***In vitro* proteasomal degradation of MBP does not require ubiquitin.** *A*, bovine MBP (*bovMBP*) ubiquitinated in the presence of HeLa cells extract (*lanes 1–5* and *8* and *9*) and BALB/c brain extract (*lanes 6* and *7*) with or without ATP, ubiquitin-aldehyde, or ubiquitin, in the indicated combinations, followed by Western blotting (*WB*) analysis (*i*). The same samples were additionally stained for polyubiquitinated proteins to verify the reaction conditions (*ii*). Artificial band existing in all MBP samples is marked by an *asterisk. B*, hydrolysis of bovine MBP monitored in a cell-free system in the presence or absence of Ub, as indicated. *C*, hydrolysis of MBP monitored in a cell-free system in the presence of either WT or methylated ubiquitin (*MeUb*). *D*, degradation of BSA, thioredoxin (*trx*), MBP, and lysozyme by the 26S proteasome in the presence of ATP (*i*). Fraction 2, which contained purified 26S proteasome and was used for *in vitro* proteolysis with activity toward the LLVY-AMC substrate (demonstrated by a native PAGE) and contained proteins related to the 19S regulatory particle (by Coomassie stain and Western blotting analysis), is shown on *panel ii. E*, plots represent rate of MBP hydrolysis by the 26S proteasome as a function of MBP concentration. *conj.*, conjugates.

We further tested whether MBP can be modified by ubiquitin *in vitro* in a reconstituted cell-free system. First, we showed that neither a HeLa extract nor a BALB/c brain extract were able to significantly ubiquitinate bovine MBP (Fig. 6*A*). Nonetheless, Western blotting analysis revealed the presence of polyubiquitinated conjugates unrelated to MBP, which confirms that the ubiquitination machinery was functional. We were able to detect traces of mono- and di-ubiquitinated MBP derivatives only in the essential presence of ubiquitin aldehyde, a specific inhibitor of ubiquitin isopeptidases. To discover a possible link between MBP ubiquitination and proteasomal degradation, we investigated the degradation of MBP in a cell-free system. In line with our previous observations, *in vitro* proteolysis of MBP in the presence of HeLa extract was independent of the presence or absence of ubiquitin (Fig. 6*B*). It is possible that the concentration of endogenous ubiquitin in HeLa extracts is sufficient to support MBP proteolysis. However, we obtained evidence that polyubiquitination is not necessary for *in vitro* MBP proteolysis because non-polymerizable methylated ubiquitin had no observable effects on the degradation of purified recombinant human MBP in the presence of BALB/c brain extract (Fig. 6*C*). To test whether MBP could be multiply mono-ubiquitinated, as in the case of the 26S-mediated processing of p105 (21), we employed a completely simplified cell-free system for *in vitro* proteolysis using a three-component mixture: MBP, ATP, and highly purified 26S proteasome isolated by two-step chromatography. Our data showed that MBP, in contrast to the other proteins, was efficiently hydrolyzed by the proteasome in the absence of any part of the ubiquitination system and ubiquitin species (Fig. 6*D*). As controls, BSA, thioredoxin, and lysozyme, the latter of which is sensitive to traces of ubiquitin, were subjected to the same conditions and showed full resistance to proteasome-mediated proteolysis under these conditions over 24 h.

**FIGURE 7.**
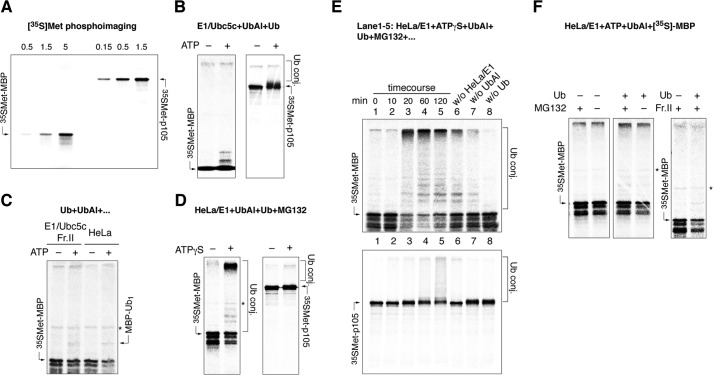
*A*, cell-free T7 TnT expression of p105 and rhMBP. The indicated amount of reaction mixture (μl) was subjected to PAGE, dried, and visualized with a PhosphorImager. *B–D*, *in vitro* translated [^35^S]Met-labeled rhMBP and p105 were incubated with E1/Ubc5c supplemented with Ub and ubiquitin aldehyde with or without ATP (*B*); E1/Ubc5c supplemented with Ub, fraction II, and ubiquitin aldehyde with or without ATP (*C*, *left*); HeLa extract supplemented with Ub and ubiquitin aldehyde with or without ATP (*C*, *right*), and HeLa extract supplemented with E1, Ub, ubiquitin aldehyde, and MG132 in the presence or absence of ATPγS. *E*, *in vitro*-translated [^35^S]Met-labeled MBP (*i*) and p105 (*ii*) ubiquitinated in a cell-free system in the presence of HeLa extract supplemented by E1, ATPγS, ubiquitin aldehyde, Ub, and MG132. The reactions were terminated at the indicated time points and subjected to PAGE (*lanes 1–5*). *Lanes 6–8* show the reactions performed for 120 min without the indicated components. *F*, degradation of *in vitro* translated [^35^S]Met-labeled rhMBP monitored in HeLa/E1-based cell-free systems supplemented with fraction II in the presence or absence of Ub and MG132, as indicated. *WB*, Western blotting; *Ub conj.*, ubiquitin conjugates; *Fr.II*, fraction II; *UbAl*, ubiquitin aldehyde.

We also found that the degradation of MBP is significantly decreased when its concentration is below 200 nm (Fig. 6*E*). A previous study demonstrated the acceleration of MBP degradation by the proteasome following ubiquitination (30). This finding may be explained by the nanomolar concentration of iodinated MBP in the reaction mixture with the 26S proteasome. To analyze the sensitivity of MBP toward proteasomal hydrolysis in the nanomolar concentration range, we employed a high-sensitivity radioactive assay. To this end, MBP and p105 were translated *in vitro* in the presence of ^35^S-labeled Met (Fig. 7*A*). MBP, at concentrations in the range of ten nanomoles per liter, was neither proteolyzed nor ubiquitinated in the presence of recombinant E1 and E2(5c) enzymes or HeLa extract (Fig. 7, *B* and *C*). To ascertain whether the absence of ubiquitination is responsible for the lack of MBP degradation, we searched for conditions for MBP polyubiquitination. We found that HeLa extract supplemented with exogenous E1 achieved polyubiquitination of MBP (Fig. 7*D*). A detailed study of this revealed that, as in the case of p105, this process is ATPγS-dependent and strictly requires the addition of exogenous ubiquitin (Fig. 7*E*). In line with this observation, the hydrolysis of MBP observed in the presence of HeLa extract supplemented with exogenous E1 shows a distinct dependence on ubiquitin (Fig. 7*F*). Thus, our data suggest that MBP is effectively degraded by the proteasome in the absence of ubiquitin only at concentrations greater than 200 nm. At lower concentrations, MBP appears to require ubiquitin to be degraded by the 26S proteasome.

##### Proteasomal Degradation of MBP Is Charge-mediated

**FIGURE 8.**
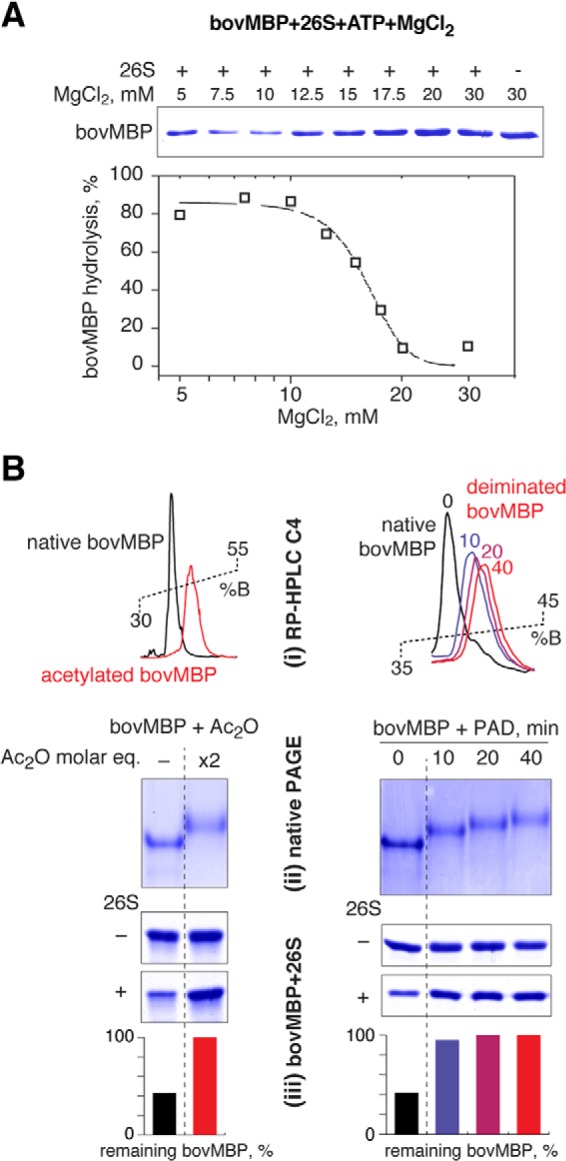
*A*, the proteasomal MBP degradation was tested in the presence of increasing concentrations of MgCl_2_, as indicated. The percentage of protein remaining was calculated as the ratio of protein at the points indicated, relative to the protein without 26S proteasome. The proteasome was active across the range of MgCl_2_ titration. *B*, acetylation (*left*) and deimination (*right*) of MBP inhibits its 26S-mediated proteolysis. Shown is an RP-HPLC profile (*i*) and native PAGE (*ii*) of the modified and non-modified MBP. *iii*, hydrolysis of the nonmodified and PAD, peptidylarginine deiminase modified MBP by the 26S proteasome, as indicated. *bovMBP*, bovine MBP.

MBP has a strikingly abnormal pI value greater than 11.5, suggesting that its hydrolysis may be charge-mediated. Thus, increasing the ionic strength should potentially inhibit its degradation. Indeed, the presence of 20 micromolar MgCl_2_ completely suppressed proteasome-mediated degradation of MBP (Fig. 8*A*). To further test the effect of decreasing the positive charge of MBP, we performed chemical acetylation and enzymatic deamination of MBP by peptidylarginine deiminase (Fig. 8*B*). The resulting increase in the retention time and the protein band shift on native PAGE gels showed a reduction in the positive charge on MBP through the blocking of lysine amino groups by acetylation or by deimination of arginine residues by peptidylarginine deiminase. Both treatments resulted in an increased resistance of MBP toward proteasomal hydrolysis.

## DISCUSSION

Importantly, our results suggest that observed ubiquitin-independent degradation of MBP by the proteasome appear to be in physiologically relevant MBP concentrations. According to a study by Barbarese and Pfeiffer (31), oligodendrocytes can accumulate MBP at the rate of 0.2 fmol per day per oligodendrocyte and reach a dynamic equilibrium of 1 fmol of MBP per cell. Together with recent data (32) that report that the volume of a mature oligodendrocyte is ∼1.7 pl, and assuming that MBP is uniformly distributed in the cell, the concentration of MBP inside an oligodendrocyte is 0.6 mm. If we consider that only 10% of this total MBP is “free” and that the remainder is membrane-associated (31), this concentration is abundantly more than that required for the ubiquitin-independent MBP proteolysis. Recently, MBP was shown to form amyloid-like fibrils (33). Thus, it is possible that free MBP is potentially highly toxic to the cell. The 26S proteasome may therefore protect oligodendrocytes from the excess of free MBP bypassing ubiquitination. Moreover, in cases of CNS inflammation or injury, oligodendrocytes will respond by compensating for the loss of MBP through the rapid accumulation of this protein and the subsequent increase in its intracellular concentration (34). Under these conditions, MBP degradation overrides the control mediated by the ubiquitination system and can be spontaneously destroyed by the proteasome. However, when the concentration of MBP returns to the nanomolar range, non-ubiquitinated MBP loses its ability to be directly recognized by the 26S proteasome.

Proteasome-mediated degradation and further presentation of myelin antigens, which recruit clonally expanded myelin-specific cytotoxic lymphocytes (35), are undoubtedly very important in the etiology and pathogenesis of MS. In their study, Antel and colleagues showed direct lysis of human oligodendrocytes by MBP-specific CD8^+^ cytotoxic lymphocytes (36). Moreover, such CD8^+^ cytotoxic lymphocytes are capable to induce experimental autoimmune encephalomyelitis in mice (11). Therefore, the observed data related to charge-mediated proteasomal MBP targeting have important physiological meaning because several post-translation modifications of the MBP molecule, including deimination, have been documented in a number of studies devoted to MS (37). Some reports suggest that a loss of charge increases the randomness and, consequently, the susceptibility of MBP toward enzymatic hydrolysis (25, 38). In contrast, our observations suggest that MBP retains its property as a proteasomal substrate only when it possesses a high positive charge. We suggest that, leading to a loss of MBP in general (39), MBP deimination, instead of ubiquitination control, may partially act as a self-protection system that prevents MBP proteolysis by the proteasome because deiminated MBP is not directly recognized by the proteasome and MBP is rapidly hydrolyzed by proteases that restrict its contact with the proteasome.

Our findings show that the ubiquitination system does not regulate MBP degradation by the proteasome. The pathophysiological importance of our findings consists in the fact that the proteasome catalytic subunits monopolistically determine spectrum and amount of MBP peptides that are further exposed on the cell surface.

## Footnotes

3 The abbreviations used are:

MBP: myelin basic protein
ODC: ornithine decarboxylase
Ub: ubiquitin
MS: multiple sclerosis
rhMBP: recombinant human MBP.
